# Emodin-Mediated Treatment of Acute Kidney Injury

**DOI:** 10.1155/2022/5699615

**Published:** 2022-03-21

**Authors:** Yu Liu, Mingquan Li, LaiKuan Teh, Liangbin Zhao, Naijing Ye, Ling Wu, Lihua Wu

**Affiliations:** ^1^Hospital of Chengdu University of Traditional Chinese Medicine, Chengdu, Sichuan, China; ^2^Department of Allied Health Sciences Faculty of Science Universiti Tunku Abdul Rahman (UTAR), Kampar, Perak, Malaysia

## Abstract

Acute kidney injury (AKI), a common condition associated with a high mortality rate, is characterized by declined glomerular filtration rate, retention of nitrogen products, and disturbances in balance of water, electrolyte, and acid-base. Up to date, there is no effective treatment for AKI. Despite the continuous improvement in blood purification techniques, a considerable proportion of patients with AKI still progress to end-stage renal disease. These patients with advanced stage of end-stage renal disease will require long-term renal replacement therapy, which places a heavy burden on the family and the society. In recent years, the use of traditional Chinese medicine (TCM) in AKI management has been gradually increasing. Clinical evidence has demonstrated that three-month treatment with TCM produced better clinical outcomes in terms of clinical effectiveness rate and improvement in renal function (serum creatinine, neutrophil gelatinase-associated lipocalin, and cystatin C) compared with Western medicine. Rhubarb is a commonly used herb in TCM for the treatment of AKI. The main active component of rhubarb is emodin, which was first recorded in Shennong's Classic of Materia Medica. It has been shown that emodin has a variety of pharmacological activities including antibacterial, anti-inflammatory, antiulcer, and immunosuppressive effects. Emodin has been found to be effective against renal fibrosis and has been widely studied for its effects on kidney diseases such as diabetic nephropathy, renal fibrosis, and AKI. Moreover, promising results have been obtained from these studies. In this study, the results obtained from research on the use of emodin for AKI treatment has been reviewed.

## 1. Introduction

Acute kidney injury (AKI) is a clinical condition featured by a remarkable decline in renal function within a short period. Patients with AKI always manifest with a decreased glomerular filtration rate, retention of nitrogen products, and disturbances in water, electrolyte, and acid-base balance [1]. An epidemiological survey showed that the incidence rate of AKI among hospitalised patients is approximately 10–15%, while the incidence rate of AKI among patients in the intensive care unit is approximately 50%. Furthermore, clinical data have indicated that AKI is one of the main causes of death in hospitalised patients [2]. In clinical settings, AKI often progresses to chronic kidney disease (CKD), which can further progress to end-stage renal disease, eventually leading to death [3]. Currently, there is no specific drug or therapy for the treatment of AKI. The current management of AKI mostly involves symptomatic treatment and renal replacement therapy. Although blood purification techniques have been improved, the mortality of patients with AKI has not been significantly reduced [4]. Recently, investigations on the treatment of AKI using traditional Chinese medicine (TCM) have become a focus of research. In a previous study, it has been found that patients with AKI presented a better renal recovery rate as well as better parameters of renal function (serum creatinine (SCr), neutrophil gelatinase-associated lipocalin, and cystatin C) following treatment with TCM preparations for three months compared with treatment with formulations used in Western medicine alone [5].

**Figure 1:**
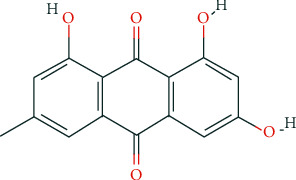
Structure of emodin.

**Figure 2:**
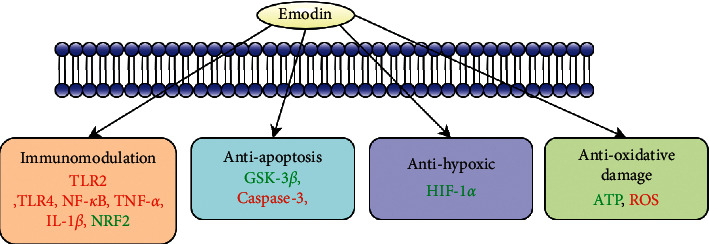
Emodin in the treatment of acute kidney injury.

Chinese rhubarb root, comprising the roots and rhizomes of *Rheum palmatum*, *Rheum tanguticum*, and *Rheum officinale,* was firstly documented in Shennong's Classic of Materia Medica. It is used for purgation during accumulation, cooling blood, haemostasis, treating blood stasis, and dredging meridians [6]. It is also commonly used to treat chronic kidney disease, diabetic nephropathy, severe acute pancreatitis (SAP), and other diseases [7–9]. Emodin is an anthraquinone derivative extracted from rhubarb. Its chemical name is 1,3,8-trihydroxy-6-methylanthraquinone (Figure 1) with the molecular formula C_15_H_10_O_5_. Results from both *in vivo* and *in vitro* experiments have revealed that emodin has biological properties, such as antibacterial [10], anti-inflammatory [11], antiulcer [12], and immunosuppressive [13] activities. Furthermore, emodin can be used to treat renal fibrosis [14]. Emodin has been extensively investigated in studies on kidney diseases, such as diabetic nephropathy [15], renal fibrosis [16], and AKI. The aim of this review was to summarize the experimental studies which examined the usage of emodin in the treatment of AKI to provide the basis for its potential in the clinical treatment of AKI. Throughout the review, we have discussed studies which showed that emodin may provide clinical benefits in AKI treatment under four different conditions (Figure 2).

## 2. Amelioration of Lipopolysaccharide-Induced AKI in Cellular Model by Emodin

AKI can result from an innate immune response that triggers an inflammation cascade, leading to a series of detrimental consequences [17–19]. It has been reported that toll-like receptors (TLRs) play an important role in the recognition of exogenous pathogens in sepsis-induced AKI. Among all TLRs, TLR2 has attracted special attention [20]. It has been shown that renal tubular epithelial cells do not respond to lipopolysaccharide, which is a key inducer of TLR2 and cytokine overexpression. Moreover, renal tubular epithelial cells release fewer cytokines in TLR2-knockout mice, suggesting that TLR2 plays an important role in the renal parenchyma and in the induction of inflammation and injury. In a previous study, emodin (20 or 40 *μ*M) has been shown to effectively protect against lipopolysaccharide-induced injury in NRK-52E cells by suppressing the expression of TLR2, nuclear factor kappa B (NF-*κ*B), and inflammatory cytokines. In addition, lipopolysaccharide-induced expression of TLR2 and NF-*κ*B in NRK-52E cells was found to be suppressed by emodin. The mRNA and protein expression of NF-*κ*B, tumour necrosis factor alpha (TNF)-*α*, interleukin (IL)-1*β*, and IL-6 were also suppressed in a dose-dependent manner by emodin. Moreover, emodin could protect renal tubular epithelial cells by inhibiting the activation of the NLRP3 inflammasome to reduce the inflammatory response and therefore alleviate lipopolysaccharide-induced AKI. However, the mechanisms underlying the suppressing effect of emodin on TLR2 expression have not been investigated [21]. A recent study also showed that the lowering levels of intercellular adhesion molecule-1 (ICAM-1), tumor necrosis factor-*α* (TNF-*α*), and fibronectin (FN) levels in the glomeruli were also involved in the protective effects of emodin against renal injury in mice model. However, whether all these factors participate in the ameliorating effects against lipopolysaccharide-induced AKI remains unclear.

## 3. Emodin in the Treatment of AKI Associated with Severe Acute Pancreatitis (SAP)

It has been shown that 15–20% of the patients with AKI develop SAP, which often leads to acute renal failure. The mortality rate of patients with acute renal failure is also estimated to be 71–84% [22]. It has been shown that the effects of emodin on AKI in patients with severe pancreatitis are mediated through various mechanisms. Firstly, Excessive TNF-*α* production, which is a cytokine mainly secreted by macrophages and adipocytes in adipose tissue, can lead to capillary damage around the glomeruli and renal tubules as well as the degeneration and necrosis of renal tubular epithelial cells. It has also been reported that the serum levels of TNF-*α*, amylase, and creatinine in rats with SAP are increased to varying degrees. In a previous study, the levels of amylase, TNF-*α*, creatinine, and injury indexes were found to be significantly lower in emodin-treated rats compared with the model group, indicating that emodin can reduce the levels of TNF-*α* and thereby alleviate renal injury in rats with SAP. In addition, pathological examination of rat kidneys under a light microscope showed that the degree of renal injury in the treatment group was lower than that in the model group at all time points, which further indicates that the protective effect of emodin against SAP and renal injury may involve blockade of all links to the mechanisms underlying intestinal mucosal barrier injury [23]. Hypoxia-inducible factor-1 alpha (HIF-1*α*) is a nuclear protein with transcriptional activity that can improve the tolerance and survival of the body in response to hypoxia. HIF-1*α* can also induce the production of a series of hypoxic responses in human body, which may correlate with regulation of the activities of tumour suppressor proteins and kidney injury molecule-1 (KIM-1) [24]. The binding of tumour suppressor proteins to HIF-1*α* is inhibited under hypoxic conditions, resulting in high expression of HIF-1*α* [25]. However, emodin can increase the protein level of HIF-1*α* in the kidney tissue by altering the formation of the HIF-1*α*-pHVL complex and inhibiting the degradation of HIF-1*α* protein, resulting in an increase in the tolerance of the kidneys to hypoxia and protection of kidney cells against hypoxic conditions [24]. KIM-1 is a type I transmembrane glycoprotein that is expressed in the kidneys of humans and rats following repair after ischemia-reperfusion injury. It has been shown that HIF-1*α* plays a role in the damage and repair of proximal renal tubular epithelial cells by regulating KIM-1 expression, thereby improving the tolerance of the kidneys to hypoxic environments [26]. In a previous study, it was found that treatment with emodin significantly increased the expression of HIF-1*α*, whereas increases in the levels of urea nitrogen and creatinine were not significant in the injury group. These findings showed that emodin can protect against kidney injury by promoting the expression of HIF-1*α* protein [24].

The key aerobic metabolic pathways that cells can adapt to in a hypoxic environment are transformed into nonoxidative carbon metabolism and adenosine triphosphate (ATP) production pathways such as glycolysis. It has been indicated that pyruvate dehydrogenase kinase (PDK) 1, which is the PDK protein encoded by the target gene of HIF-1*α*, inhibits the synthesis of acetyl-CoA, blocks the tricarboxylic acid cycle, and reduces oxygen consumption [27]. Consequently, under hypoxic conditions, ATP production in tissues decreases along with the decreased expression of HIF-1*α*, leading to the generation of more oxygen free radicals and making cells more susceptible to apoptosis. Furthermore, emodin can improve the ability of kidney cells to tolerate hypoxia by enabling the reestablishment of metabolic pathways in kidney cells, thereby alleviating the damaging effects of oxygen free radicals and endotoxin on kidney cells and preventing apoptosis and necrosis of kidney cells [24].

Glycogen synthase kinase-3*β* (GSK-3*β*) is a multifunctional serine/threonine protein kinase in eukaryotic cells that plays a role in necrosis and organ failure in inflammation-related diseases. It has also been reported that GSK-3*β* may be involved in signalling number of cellular signalling pathways. In a previous *in vivo* study with 60 rats, it was found that emodin upregulated the expression of GSK-3*β* and maintained the levels of SCr and blood urea nitrogen (BUN) within normal ranges. Therefore, it is reasonable to believe that emodin can upregulate GSK-3*β* expression to improve the ability of renal cells to withstand hypoxia which contribute to the protection of emodin against renal injury in SAP [28].

## 4. Emodin for Treating Ischemia-Reperfusion-Induced AKI

Temporal blockage of the renal arteries in renal surgeries can inevitably lead to the development of renal ischemia-reperfusion injury, which is one of the major causes of postoperative acute renal injury. It has been reported that postoperative acute renal injury involves proinflammatory reactions (such as inflammatory response), oxidative stress, and metabolic disorders. Some studies have shown that ischemia-reperfusion can significantly enhance apoptosis and worsen renal function and tissue injury in rats. In addition, pretreatment with emodin was found to result in a significant reduction in the inflammatory response following renal ischemia-reperfusion injury, leading to reduced apoptosis and improved renal function. Furthermore, emodin upregulates Nrf2 expression and downregulates the TLR4/NF-*κ*B signalling pathway. Through modulating these signalling pathways, emodin reduces oxidative stress and caspase-3 activity and exerts anti-inflammatory and antiapoptotic effects. These findings show that emodin can produce therapeutic effect in ischemia-reperfusion-induced AKI [29].

## 5. Effects of Emodin on Intestinal Flora

Intestinal flora refer to parasitic microbes in the human intestines which play important physiological roles in metabolism and immune response. Patients with kidney disease often present with intestinal flora imbalances, increased intestinal mucosal permeability, and increased level of toxin absorption into the blood, which can lead to systemic inflammation and aggravation of renal injury. Moreover, large amounts of urea nitrogen, creatinine, and uric acid cannot be eliminated from the body timely, resulting in further deterioration of the renal function. Jiang et al. found an imbalance in the amount and ratio of flora, a decrease in the number of beneficial bacteria, and an increase in the number of harmful bacteria in the model group, which were established by intraperitoneal injection of small dose of gentamicin sulphate in rats for 7 days. However, these were not found in the normal group. Furthermore, the number of harmful bacteria was found to positively correlate with BUN values and creatinine concentrations. It has been shown that in patients with acute renal injury, the intestine replaces the kidney as the largest organ for protein metabolism. Other nonprotein nitrogenous compounds excreted through the intestine provide conditions for the growth and reproduction of bacteria. Moreover, it was found that the levels of D-lactic acid and plasma endotoxin in the serum were significantly increased in model rats. In rat models with AKI, the intestinal mucosa is damaged, whereas intestinal permeability is increased. Consequently, toxic substances secreted by harmful bacteria can easily enter the circulatory system and result in the development of enterogenous endotoxemia. The results of this study also showed that emodin regulated intestinal flora, normalised the proportion of flora, reduced the accumulation of enterogenous urotoxin in the blood, accelerated the clearance of enterogenous urotoxin, alleviated intestinal mucosal injury, and improved the renal function in rats with AKI [30].

## 6. Limitations of Use

Pharmacokinetic experiments discovered that emodin is rapidly metabolized by the liver via glucuronidation phase 2 metabolisms across multiple dose levels. As a result, poor oral bioavailability and ADME does not make the oral route a promising route of administration. More detailed of understanding of emodin is needed to improve its bioavailability. Attempts to inhibit glucuronidation metabolism and increase absorption have been underway using 2,3,5,4′-tetrahydroxystilbene-2-O-*β*-D-glucoside (TSG). Likewise, piperine has been found to improve pharmacokinetic properties including the maximum plasma concentration and area under the curve for isolated emodin [12]. However, more data on the pharmacokinetics of emodin need to be collected to determine the appropriate dosage for clinical usage.

## 7. Conclusions

The pathogenesis of AKI involves inflammation, oxidative stress, apoptosis, ischemia-reperfusion, and intestinal flora imbalance. Emodin may provide clinical benefits in the treatment of AKI by exerting inhibitory effects against inflammation, oxidative stress, and apoptosis. In addition, emodin can reduce of enterogenous toxins and accelerate the clearance of enterogenous toxins. Although studies have shown the effectiveness of emodin in the treatment of AKI, a few issues need to be further investigated. Firstly, all the *in vivo* studies were conducted in animals. Therefore, clinical evidence needs to be collected to provide evidence for its potential use in clinical settings. Secondly, the optimal intervention period for emodin is unclear, even though emodin exhibits preventive and therapeutic effects against AKI. Thirdly, the pathogenesis of AKI is complex, which involves a few pathways and signalling molecules. Consequently, single-target interventions may not able to produce beneficial clinical outcomes. Effective components of TCM herbs can act on the body through multiple pathways and targets. Therefore, the molecular mechanisms underlying the therapeutic effect of emodin in AKI must be studied using modern molecular biology. It is expected that after further experimental and clinical research, emodin may be considered a potential therapeutic drug for the treatment of AKI to improve the clinical outcome in terms of reducing mortality associated with AKI as well as preventing the progression to end-stage renal disease.

## Data Availability

The datasets supporting the conclusions of this article are available in the PubMed (https://www.ncbi.nlm.nih.gov/) and CNKI (https://www.cnki.net/) databases.

## Abbreviations

AKI: Acute kidney injury
CKD: Chronic kidney disease
SAP: Severe acute pancreatitis
ESRD: End-stage renal disease
HIF-1*α*: Hypoxia-inducible factor-1*α*
GSK-3*β*: Glycogen synthase kinase-3*β*
TLR2: Toll-like receptor 2.

## References

[1] Ronco C., Bellomo R., Kellum J. A. (2019). Acute kidney injury. *The Lancet*.

[2] Al-Jaghbeer M., Dealmeida D., Bilderback A., Ambrosino R., Kellum J. A. (2018). Clinical decision support for in-hospital AKI. *Journal of the American Society of Nephrology*.

[3] Zuk A., Bonventre J. V. (2016). Acute kidney injury. *Annual Review of Medicine*.

[4] Brix S, Stahl R (2017). Acute kidney injury. *Deutsche Medizinische Wochenschrift*.

[5] Fengbo Z., Yongzhi L., Jianguo Z., Jiawang G. (2018). Observation on the curative effect of activating blood circulation and removing blood stasis on acute kidney injury in patients with nephropathy during hospitalization. *Chinese Journal of Integrated Traditional and Western Nephrology*.

[6] Xiang H., Zuo J., Guo F., Dong D. (2020). What we already know about rhubarb: a comprehensive review. *Chinese Medicine*.

[7] Wang H., Song H., Yue J., Li J., Hou Y. B., Deng J. L. (2012). Rheum officinale (a traditional Chinese medicine) for chronic kidney disease. *Cochrane Database of Systematic Reviews*.

[8] Gu L., Wan Y., Wan M. (2003). Advances in the study on molecular mechanism of diabetic nephropathy treated with Rheum officinale. *Zhongguo Zhongyao Zazhi*.

[9] Chen X., Yang K., Jing G., Yang J., Li K. (2020). Meta‐analysis of efficacy of rhubarb combined with early enteral nutrition for the treatment of severe acute pancreatitis. *Journal of Parenteral and Enteral Nutrition*.

[10] Dey D., Ray R., Hazra B. (2014). Antitubercular and antibacterial activity of quinonoid natural products against multi-drug resistant clinical isolates. *Phytotherapy Research*.

[11] Gao J., Wang F., Wang W., Su Z., Guo C., Cao S. (2014). Emodin suppresses hyperglycemia-induced proliferation and fibronectin expression in mesangial cells via inhibiting cFLIP. *PLoS One*.

[12] Dong X., Fu J., Yin X. (2016). Emodin: a review of its pharmacology, toxicity and pharmacokinetics. *Phytotherapy Research*.

[13] Qu K., Shen N.-Y., Xu X.-S. (2013). Emodin induces human T cell apoptosis in vitro by ROS-mediated endoplasmic reticulum stress and mitochondrial dysfunction. *Acta Pharmacologica Sinica*.

[14] Yang F., Deng L., Li J. (2020). Emodin retarded renal fibrosis through regulating HGF and TGF*β*-smad signaling pathway. *Drug Design, Development and Therapy*.

[15] Tian N., Gao Y., Wang X. (2018). Emodin mitigates podocytes apoptosis induced by endoplasmic reticulum stress through the inhibition of the PERK pathway in diabetic nephropathy. *Drug Design, Development and Therapy*.

[16] Xu L., Gao J., Huang D. (2021). Emodin ameliorates tubulointerstitial fibrosis in obstructed kidneys by inhibiting EZH2. *Biochemical and Biophysical Research Communications*.

[17] Akira S., Uematsu S., Takeuchi O. (2006). Pathogen recognition and innate immunity. *Cell*.

[18] Lee H. K., Iwasaki A. (2007). Innate control of adaptive immunity: dendritic cells and beyond. *Seminars in Immunology*.

[19] Zager R. A., Johnson A. C. M., Lund S., Randolph-Habecker J. (2007). Toll-like receptor (TLR4) shedding and depletion: acute proximal tubular cell responses to hypoxic and toxic injury. *American Journal of Physiology-Renal Physiology*.

[20] Werts C., Tapping R. I., Mathison J. C. (2001). Leptospiral lipopolysaccharide activates cells through a TLR2-dependent mechanism. *Nature Immunology*.

[21] Li Y., Xiong W., Yang J. (2015). Attenuation of inflammation by emodin in lipopolysaccharide-induced acute kidney injury via inhibition of toll-like receptor 2 signal pathway. *Iranian journal of kidney diseases*.

[22] Kunieda T., Minamino T., Nishi J.-i. (2006). Angiotensin II induces premature senescence of vascular smooth muscle cells and accelerates the development of atherosclerosis via a p21-dependent pathway. *Circulation*.

[23] Wang P. (2012). Study on the protection of Emodin on kidney damage in rat with severe acute pancreatitis. *Modern Journal of Integrated Traditional Chinese and western Medicine*.

[24] Zhi-ling L. I., Zhang D., Jiang-wei L. I. U., Wang H. (2015). Effects of emodin on the expression of hypoxia inducible factor-1*α* protein in rats with severe acute pancreatitis-associated renal injury. *West China Medical Journal*.

[25] Haase V. H. (2006). Hypoxia-inducible factors in the kidney. *American Journal of Physiology-Renal Physiology*.

[26] Huo W., Jin F., Li Q. (2011). Regulation of renal injury molecule-1 expression signal pathway in renal tubular hypoxia injury. *Journal of renal disease and dialysis, renal transplantation*.

[27] Wang P., Kong F., Chen X. (2011). HIF-1 signaling pathway in hypoxic cell stress. *Journal of Zhejiang University: Medical Edition*.

[28] Wen-shuai L. I. U. (2017). Effect of emodin on the levels of hypoxia inducible factor-l*α*and glycogen synthase kinase-3*β*in rats with severe acute pancreatitis. *The Chinese Journal of Clinical Pharmacology*.

[29] Long L. Emodin attenuates IRI induced inflammation and oxidative stress in rat kidney through Nrf2/TLR4/NF-*κ* B Signaling Pathway. *China Academic Journal Electronic Publishing House*.

[30] Sun J., Luo J. W., Yao W. J., Luo X. T., Su C. L., Wei Y. H. (2019). Effect of emodin on gut microbiota of rats with acute kidney failure. *Zhongguo Zhongyao Zazhi*.

